# Do chromatin changes around a nascent double strand DNA break spread spherically into linearly non-adjacent chromatin?

**DOI:** 10.3389/fgene.2013.00139

**Published:** 2013-07-19

**Authors:** Velibor Savic

**Affiliations:** ^1^Department of Clinical Medicine, Brighton-Sussex Medical School, University of SussexBrighton, UK; ^2^Genome Damage and Stability Centre, University of SussexBrighton, UK

**Keywords:** DNA damage, double strand DNA break, chromatin, DNA damage response, gamma-H2AX, ATM, RNF168

## Abstract

In the last decade, a lot has been done in elucidating the sequence of events that occur at the nascent double strand DNA break. Nevertheless, the overall structure formed by the DNA damage response (DDR) factors around the break site, the repair focus, remains poorly understood. Although most of the data presented so far only address events that occur in chromatin *in cis* around the break, there are strong indications that in mammalian systems it may also occur *in trans*, analogous to the recent findings showing this if budding yeast. There have been attempts to address the issue but the final proof is still missing due to lack of a proper experimental system. If found to be true, the spatial distribution of DDR factors would have a major impact on the neighboring chromatin both *in cis* and *in trans*, significantly affecting local chromatin function; gene transcription and potentially other functions.

## INTRODUCTION

Double strand DNA breaks (DSBs) are one of the most dangerous genetic lesions the DNA can incur. They can occur exogenously or endogenously; they are mostly random but can also be programed as a part of a wider biological process (mating type switching in yeast, meiotic recombination, V(D)J recombination and class-switch recombination in mammals). They can be repaired through two complementary pathways: the faster but potentially error-prone non-homologous end joining (NHEJ) and through slower and more precise homologous recombination (HR). These pathways are to a large extent conserved from yeast to humans, but the repair pathway of choice can vary between species – from predominantly HR-mediated repair in yeast to predominantly NHEJ in humans. Notably, if left unattended, such DSBs can induce cell death while their improper repair can result in alterations of the genetic makeup of the cell, in higher organisms potentially leading to oncogenic transformation.

Double strand DNA breaks induction and subsequent repair does not occur in isolation, but in the context of a nucleoprotein superstructure called chromatin. Chromatin is a highly repetitive structure based on the 146 bp of DNA wound around a histone octamer, together termed a nucleosome, and connected via linker DNA of varying length. This creates a bead-on-a-string structure which can be compacted further, resulting in overall DNA compaction of up to a 100,000-fold in order to fit into the nucleus. Such high compaction necessitates an organized structure within the DNA to allow for proper function and prevent entanglement. There are varying levels of compaction of specific genomic regions (reviewed in Gilbert et al., 2005), depending on the association of additional proteins to histones.

Structural differences in chromatin compaction frequently reflect the function of various chromatin regions. Less compacted euchromatin regions tend to be more transcriptionally active and the more compacted heterochromatin ones predominantly silent. Moreover, heterochromatin tends to cluster in the center of a chromosome territory, while euchromatin forms the periphery and the interchromosomal interaction surfaces (reviewed in Gilbert et al., 2005). This creates a significant potential for physical proximity between linearly distant intrachromosomal regions, or between regions on different chromosomes. Mostly it’s passive proximity through folding and compaction, but some may be both active and targeted, like the concerted interaction of genes from different chromosomes: for better response to signaling (Spilianakis et al., 2005), for regulated (Lomvardas et al., 2006) or concerted transcription (Lin et al., 2009).

Complex chromatin structure creates a significant challenge in both recognition and repair of DSBs. A nascent break needs to be sensed and the repair machinery needs to have access to the broken ends. Most DSBs are sensed and repaired very fast via NHEJ, but a fraction of them persists and requires the activation of the full DNA damage response (DDR) signaling. This assists the repair mechanisms in preventing separation of broken ends (Bredemeyer et al., 2008), helps avoid promiscuous repair and prevents DNA replication or mitosis to initiate prior to physical repair (reviewed in Ciccia and Elledge, 2010).

## ATM ACTIVATION AT THE ONSET OF DDR SIGNALING

One of the earliest events in the activation of DDR signaling is activation of the ataxia telangiectasia-mutated (ATM) kinase. When inactive, ATM exists as a homodimer that dissociates upon activation, creating two enzymatically active monomers (Bakkenist and Kastan, 2003). Active ATM can phosphorylate numerous targets, both located proximally to the break site and inducing focal accumulation of DDR factors (together termed an IRIF – ionizing radiation-induced focus) or dispersed throughout the nucleus (Matsuoka et al., 2007). Activation is concomitant with phosphorylation of the monomer at several sites, including S1981 (subsequently termed pATM). The phosphorylation occurs through cross-phosphorylation within the ATM dimer, and in the case of humans it is required for dimer dissociation and activation (Bakkenist and Kastan, 2003). The implication is that individual inactive dimers are independently activated and that the phosphorylation is not exogenous, as previously hypothesized (Kitagawa and Kastan, 2005).

What then activates ATM and where in the nucleus does it occur? The evidence suggests that it could be through the interaction with the deprotected DNA at the site of a DSB. ATM is activated by nascent DSBs, and ChIP analyses have shown that pATM accumulates in the proximity of a break site (Berkovich et al., 2007). One of the earliest events around the break site in human cells is probably nucleosome removal and creation of a longer stretch of naked DNA, analogous to yeast (Tsukuda et al., 2005). Notably, naked DNA in excess of 200 bp is enough to activate ATM both *in vitro* and in *Xenopus* extracts (You et al., 2007), even in the absence of deprotected DNA ends. The MRN complex (Mre11/Rad50/NBS1), one of the earliest DSB sensors which binds directly to the break site (reviewed in Stracker and Petrini, 2011), binds also ATM and is required for its full activation, potentially through facilitating the interaction of ATM with the DNA. In contrast, globally chromatin relaxation through trichostatin A (TSA) or chloroquine treatment, activates ATM throughout the nucleus without forming foci (Bakkenist and Kastan, 2003). Thus, it is the localized chromatin relaxation around a nascent DSB what induces the site-specific ATM activation.

Upon activation, ATM phosphorylates numerous targets, including a histone H2A variant H2AX (Burma et al., 2001). H2AX is highly abundant in the cells and comprises 5–25% of the total nuclear H2A (Rogakou et al., 1998, 1999). It differs from the canonical H2A in having an extended C-terminus where it becomes phosphorylated on serine 139 during DDR, forming γH2AX. This initiates very early upon break induction, within seconds, and at equilibrium γH2AX region can extend up to 500 kb linearly away from the break site (Meier et al., 2007; Savic et al., 2009). γH2AX serves as the earliest histone mark which specifies the region in chromatin where a DNA break occurred. In its absence, downstream events like MDC1 (mediator of DNA damage checkpoint 1) binding, RNF168 accumulation or 53BP1 foci formation do not occur properly. This results in DNA damaging sensitivity and illustrates the importance of local chromatin in the proper repair of DNA breaks. Notably, not only cells lacking γH2AX (H2AX S139A) show DNA damage hypersensitivity, but the mutants overexpressing phosphomimetic S139E as well (Celeste et al., 2003a,b), even though H2AX S139E can constitutively activate DDR signaling (Kobayashi et al., 2009). This suggests that it is the absence of local accumulation what is impairing DNA repair in H2AX S139E, and not any reduction in global DDR signaling.

## MAKING THE CASE FOR THE FORMATION OF γH2AX AND K63-UBIQUITYLATION IN 3D

γH2AX recruits MDC1 which in turn binds activated ATM and retains it near the break site (Stucki and Jackson, 2006; Berkovich et al., 2007; So et al., 2009). This has led to the hypothesis that the γH2AX-dependent recruitment of MDC1 and pATM creates a feed-forward mechanism that leads to an extended γH2AX region (Stucki and Jackson, 2006). Subsequent results have confirmed that in the absence of ATM γH2AX levels are reduced in both extent and density (Savic et al., 2009). However, the absence of MDC1 had no effect on the extent of the γH2AX-containing region even though it reduced the peak intensity to the ATM^-^^/^^-^ levels, indicating that only the high, proximal γH2AX levels are dependent on MDC1 anchoring pATM on chromatin and that the distal γH2AX is independent of this mechanism (Savic et al., 2009).

The question that arises is how is this distal γH2AX then formed? As mentioned, ATM activation is probably site specific and it occurs at the DSB, but many of the ATM targets do not localize to the break site. Thus, a fraction of the activated ATM has to diffuse from the break site and phosphorylate targets throughout the nucleus. The resulting concentration gradient of active pATM molecules could be the defining factor in determining the γH2AX spread. Distribution of pATM upon laser stripe-mediated DNA damage induction indicates an initial pATM accumulation at the stripe subsequently followed by the overall increase in the pATM signal throughout the nucleus, fitting with the idea of localized activation followed by diffusion (Kruhlak et al., 2006). Notably, when chromatin is globally induced to relax through TSA or chloroquine treatment, ATM is activated globally, without forming foci (Bakkenist and Kastan, 2003). Thus, the site-specific changes in chromatin around nascent DSBs are what induces site-specific ATM activation.

**FIGURE 1:**
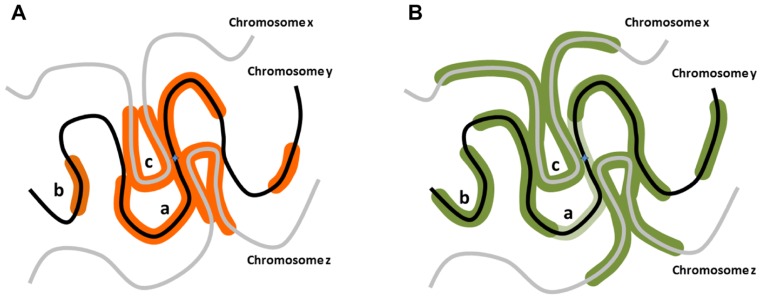
**Potential distribution of DNA damage associated modifications around nascent DSBs.** **(A)** When a DSB occurs on a chromosome y, aside from the confirmed linear phosphorylation in the vicinity of the break site along the chromosome y (a), H2AX could be phosphorylated on distal chromosomal regions of the same chromosome (b), or on regions of different chromosomes (chromosomes x, z) in the vicinity of the break site (c). **(B)** RNF168 polyubiquitylation-dependent 53BP1 distribution could exhibit distribution analogous to γH2AX, but potentially more expanded distally from the break site. Notably, in G2 stage of the cell cycle in particular, 53BP1 distribution pattern may be only partially overlapping with the γH2AX region as it is excluded from the vicinity of the break site bound by BRCA1 (light green; Chapman et al., 2012).

Diffusible pATM as the generator of distal γH2AX would indicate that such a chromatin mark can be deposited non-linearly and does not require tracking along the DNA fiber (**Figure 1A**). In fact, γH2AX formation in human cells at unprotected telomeres can form discontinuously (Meier et al., 2007). Although the mechanisms are somewhat different, it is also of note that in budding yeast the H2A phosphorylation equivalent to γH2AX (γH2A) can skip over heterochromatic regions (Kim et al., 2007), supporting the idea that the γH2AX spreading may not occur through chromatin tracking.

pATM diffusion hypothesis suggests that at high enough concentration, pATM may even phosphorylate H2AX and generate the DDR cascade independently of the break site, analogous to the skipping of chromatin regions (**Figure 1A**). Notably, artificially high concentrations of NBS1 or Mre11 can lead to activation of the downstream DDR cascade and formation of an IRIF even on undamaged chromatin (Soutoglou and Misteli, 2008). The reason may be that Mre11 and NBS1 can bind and recruit non-phosphorylated ATM to damaged chromatin (So et al., 2009), thus bringing ATM in close proximity to the DNA which may be enough to activate it (You et al., 2007). The artificial accumulation of ATM itself was enough to activate the initial damage response and γH2AX formation, but curiously did not elicit a more downstream 53BP1 accumulation (Stewart et al., 2003).The intriguing possibility is that chromatin anchoring of ATM (by means of LacO/LacI-ATM association) is not enough to trigger 53BP1 focus formation, but the endogenous ATM, initially recruited and activated by chromatin-associated MRN and subsequently diffusing away from the break, is what elicits 53BP1 foci.

RNF168, ubiquitin ligase functioning downstream of ATM in DDR, displays properties similar to the ATM in the γH2AX spatial distribution hypothesis. It is a ubiquitin ligase that creates lysine 63-linked polyubiquitin chains focally around the DSBs in chromatin, among others on histone H2A (Doil et al., 2009; Stewart et al., 2009; Panier et al., 2012). This is dependent on the preceding H2A monoubiquitylation via RNF8, which in turn is dependent on MDC1 and serves as an anchor and a primer for the polyubiquitin assembly (Doil et al., 2009; Stewart et al., 2009). Although true for initial recruitment and activity, subsequent ubiquitylation of chromatin seems independent of RNF8, thus it appears that RNF168 has an autoregulatory effect on its own chromatin recruitment and signal amplification capacity. This would indicate that the major way RNF168 is regulated at break sites is through overall availability, which is exactly what a recent study showed (Gudjonsson et al., 2012). There, the size of individual 53BP1 foci formed around a site-specific breaks was increased in the cells lacking TRIP12 and UBR5, the two E3 ubiquitin ligases which regulate RNF168 turnover. Moreover, the incremental increase in the ionizing radiation gradually reduced the size of 53BP1 foci in TRIP12^siRNA^UBR5^siRNA^ cells, but the foci were nonetheless larger than in equivalent controls, suggesting RNF168 enzyme availability is a factor regulating 53BP1 focus size.

The proposed mechanism through which RNF168 could lead to spatial ubiquitylation and 53BP1 recruitment is somewhat similar to the way γH2AX is induced by ATM activity (**Figure 1B**). RNF168 association with chromatin requires chromatin to be primed through RNF8-mediated ubiquitylation of histone H2A, thus creating the binding sites for RNF168 (Panier et al., 2012). Similarly, ATM recruitment to chromatin requires prior priming through γH2AX induction which creates MDC1 binding sites (Stucki and Jackson, 2006). In contrast to ATM, which can serve as its own priming enzyme, RNF8 is essential for RNF168 function at break sites (Doil et al., 2009; Stewart et al., 2009). Subsequent to initial binding, RNF168 may extend the monoubiquitin tag of the binding site but may also be able to polyubiquitylate the neighboring nucleosomes and create new binding sites irrespective of RNF8 (Panier et al., 2012). Crucially, the RNF168-mediated ubiquitylation has not been shown to have any DNA tracking ability, thus it may depend on the proximity of the substrate nucleosomes. This creates a potential for a feed-forward mechanism and signal to jump between chromatin regions, if the latter is looped close to the original source of signaling. The ability of 53BP1 foci to grow significantly larger than to γH2AX-containing seeding region, proportionally to the amount of available RNF168 in the cell (Gudjonsson et al., 2012), strongly supports the idea of RNF168-mediated ubiquitylation spreads beyond the confines of γH2AX-coated chromatin region (**Figure 1B**).

Chapman et al. (2012) go even further and show that 53BP1 foci preferentially form outside their γH2AX seeding regions. This is crucial structural evidence which shows that the predominant way RNF168 induces 53BP1 binding is not through inducing ubiquitylation within the region where RNF8 creates the seeding monoubiquitylation, but in the region that results from positive autoregulatory signals beyond it. Moreover, they report non-random changes in the structure of such 53BP1 foci, depending on the cell cycle stage where the DSBs are induced and on the central presence of BRCA1. This indicates that IRIF formation, at least at the level of 53BP1 binding, may indeed be three dimensional, forming a regulatable sphere around a putative γH2AX or BRCA1-demarcated break site. Unfortunately, due to the constraints of the immunofluorescent analysis, it is impossible to say whether the resultant globular structure includes 53BP1 coating non-linear regions of chromatin or is the result of a specific folding structure of the linearly adjacent chromatin loops.

## THE CONUNDRUM

There was at least one attempt to answer the question of spatial effect a DSB on chromatin (Iacovoni et al., 2010). The ChIP-Seq analysis in a human cell line showed no apparent spatial γH2AX spread, since there was no detectable γH2AX accumulation outside of the linear chromatin regions harboring target sequences for enzymatically induced DSB. Unfortunately, this conclusion is based on a premise that chromatin is static and that in every cell in the analyzed population the same distal, inter- or intrachromosomal regions will fold back to be in close proximity to the break site. In contrast, there is a large body of evidence which shows that the position and interaction of chromatin regions is stochastic and in most cases with only moderate tendencies of association with a specific partner on a population level (reviewed in Gilbert et al., 2005), far below the requirements to support the starting premise above. The nuclear position of a genomic locus is also not fixed and a the locus can move within a confined space over time (reviewed in Dion and Gasser, 2013), potentially leading to stochastic interactions with neighboring chromatin. Moreover, the presence of a DSB seems to increase the average mobility of neighboring chromatin in mammals (Krawczyk et al., 2012), further reducing the chance of interchromosomal or interregional chromatin interactions being uniform throughout the population, implied in the conclusions by Iacovoni et al. (2010). On the other hand, if the spatial distribution hypothesis is valid, the resulting γH2AX phosphorylation would spread stochastically throughout the genome, in accordance with the likelihood of association with different chromatin regions of the break site. In this case, the result would be a change in the ChIP-Seq signal far below the measurable level, fitting with the primary data.

Given the problem of stochastic interactions, the only way to practically address this issue is to use a cellular system where the association of the break site and a distal chromatin region is inducible and non-random. This creates a problem, as only a handful of specific interchromosomal interactions have been described in mammalian cells, and none include a site specific, inducible DSB. However, two recent reports using system of site-specific break coupled with a defined homologous donor site shed a new light and show that γH2A can exhibit discontinuous, both intra- and interchromosomal distribution (Li et al., 2012; Renkawitz et al., 2013). In one report, Li et al. (2012) show that upon HO endonuclease-mediated break induction and activation of the HR repair mechanism, RAD51 can interact not only with the broken DNA fragment but also the homologous donor sequence on a separate chromosome as well. In the second report, Renkawitz et al. (2013) show a comparable result with the donor distally on the same chromosome. Furthermore, they showed that direct physical association during strand invasion in HR is not necessary for *in trans* γH2A induction. In yeast, chromosome centromeres tend to be clustered and in close proximity, and they have shown that upon DSB induction in the centromeric region of chromosome IV, centromeres on chromosomes XI and XVI also become positive for γH2A and RAD51, even in the absence of a homology donor. Crucially, these signals are not detectable if the DSB is induced more distally and not in the centromeric region. Thus, in yeast γH2A phosphorylation can spread *in trans* to unbroken DNA in close proximity and may not require direct physical interaction.

The Jentsch group has previously reported starkly different Rad51 findings using a similar system of a site-specific DSB, but in cells without a specific donor sequence (Kalocsay et al., 2009). Here they showed increased Rad51 signal to be exclusively intrachromosomal, non-specific and to varying degrees along the whole chromosome. However, as this system lacks a homology donor and no productive strand invasion can take place, Rad51 distribution is most probably the just a result of random homology searches. Generally, the constraints on chromatin movement result in intrachromosomal interactions being much more prevalent than interchromosomal (Lieberman-Aiden et al., 2009). In mouse lymphocytes similar constraints on broken DNA ends determined the choice of translocation partners to such an extent that intrachromosomal translocations per megabase are at least an order of magnitude more frequent than the ones across chromosomes (Zhang et al., 2012). In the case of Kalocsay et al. (2009), the same preferences probably masked the low level cross-chromosome interactions in favor of the more prominent intrachromosomal ones. The stark difference between the two reports from the same group clearly showcases how essential a targeted system is in properly addressing the *in trans* effects around a DSB and why a targeted system is critical to properly address this question in mammals.

## THE EFFECTS OF 3D SPREAD

What would be the consequence of a three dimensional IRIF? Two recent studies have shown that a nascent DSB induces transcriptional silencing of a gene *in cis* (Shanbhag et al., 2010; Pankotai et al., 2012). Moreover, Shanbhag et al. (2010) have shown that this repression does not depend on the physical presence of a break, as ATM inhibition upon DSB induction abolishes transcriptional repression. It does, however, depend on the downstream DDR signaling, including γH2AX induction and in particular subsequent RNF168 mediated ubiquitylation. If γH2AX and polyubiquitylation through RNF168 can indeed extend beyond the linear DNA, aside from resulting in the described transcriptional inhibition could occur *in trans* as well as* in cis*. This would have a major impact on chromatin function – not only would it result in a spatial transcriptional silencing, fitting with the described overlap of 53BP1 foci and zones of reduced mRNA synthesis (Gudjonsson et al., 2012), but the changes could be even more profound and affect other chromatin functions as well.

## CONCLUSION

Even though in the last decade our knowledge about the interaction networks governing the DDR in mammals has grown exponentially, the spatial organization of this network, in particular the spatial organization of an IRIF is still a mystery. Initial attempts have shown great promise and indicate some form of spatial regulation of the DDR, but the question whether DDR factors indeed accumulate in a non-linear fashion is still left unanswered. The implications of such an accumulation on the structure and function of the adjacent chromatin are many, but addressing them directly and unequivocally will have to await the development of specific, targeted approach.

## Conflict of Interest Statement

The author declares that the research was conducted in the absence of any commercial or financial relationships that could be construed as a potential conflict of interest.

## References

[B1] Bakkenist C. J. Kastan M. B. (2003). DNA damage activates ATM through intermolecular autophosphorylation and dimer dissociation. *Nature* 421 499–506 10.1038/nature01368 12556884

[B2] Berkovich E. Monnat R. J. Jr. Kastan M. B. (2007). Roles of ATM and NBS1 in chromatin structure modulation and DNA double-strand break repair. *Nat. Cell Biol.* 9 683–690 10.1038/ncb1599 17486112

[B3] Bredemeyer A. L. Helmink B. A. Innes C. L. Calderon B. McGinnis L. M. Mahowald G. K. (2008). DNA double-strand breaks activate a multi-functional genetic program in developing lymphocytes. *Nature* 456 819–823 10.1038/nature07392 18849970 PMC2605662

[B4] Burma S. Chen B. P. Murphy M. Kurimasa A. Chen D. J. (2001). ATM phosphorylates histone H2AX in response to DNA double-strand breaks. *J. Biol. Chem.* 276 42462–42467 10.1074/jbc.C100466200 11571274

[B5] Celeste A. Difilippantonio S. Difilippantonio M. J. Fernandez-Capetillo O. Pilch D. R. Sedelnikova O. A. (2003a). H2AX haploinsufficiency modifies genomic stability and tumor susceptibility. *Cell* 114 371–383 10.1016/S0092-8674(03)00567-1 12914701 PMC4737479

[B6] Celeste A. Fernandez-Capetillo O. Kruhlak M. J. Pilch D. R. Staudt D. W. Lee A. (2003b). Histone H2AX phosphorylation is dispensable for the initial recognition of DNA breaks. *Nat. Cell Biol.* 5 675–679 10.1038/ncb1004 12792649

[B7] Chapman J. R. Sossick A. J. Boulton S. J. Jackson S. P. (2012). BRCA1-associated exclusion of 53BP1 from DNA damage sites underlies temporal control of DNA repair. *J. Cell Sci.* 125 3529–3534 10.1242/jcs.105353 22553214 PMC3445322

[B8] Ciccia A. Elledge S. J. (2010). The DNA damage response: making it safe to play with knives. *Mol. Cell* 40 179–204 10.1016/j.molcel.2010.09.019 20965415 PMC2988877

[B9] Dion V. Gasser S. M. (2013). Chromatin movement in the maintenance of genome stability. *Cell* 152 1355–1364 10.1016/j.cell.2013.02.010 23498942

[B10] Doil C. Mailand N. Bekker-Jensen S. Menard P. Larsen D. H. Pepperkok R. (2009). RNF168 binds and amplifies ubiquitin conjugates on damaged chromosomes to allow accumulation of repair proteins. *Cell* 136 435–446 10.1016/j.cell.2008.12.041 19203579

[B11] Gilbert N. Gilchrist S. Bickmore W. (2005). Chromatin organization in the mammalian nucleus. *Int. Rev. Cytol.* 242 283–336 10.1016/S0074-7696(04)42007-5 15598472

[B12] Gudjonsson T. Altmeyer M. Savic V. Toledo L. Dinant C. Grofte M. (2012). TRIP12 and UBR5 suppress spreading of chromatin ubiquitylation at damaged chromosomes. *Cell* 150 697–709 10.1016/j.cell.2012.06.039 22884692

[B13] Iacovoni J. S. Caron P. Lassadi I. Nicolas E. Massip L. Trouche D. (2010). High-resolution profiling of gammaH2AX around DNA double strand breaks in the mammalian genome. *EMBO J.* 29 1446–1457 10.1038/emboj.2010.38 20360682 PMC2868577

[B14] Kalocsay M. Hiller N. J. Jentsch S. (2009). Chromosome-wide Rad51 spreading and SUMO-H2A.Z-dependent chromosome fixation in response to a persistent DNA double-strand break. *Mol. Cell* 33 335–343 10.1016/j.molcel.2009.01.016 19217407

[B15] Kim J. A. Kruhlak M. Dotiwala F. Nussenzweig A. Haber J. E. (2007). Heterochromatin is refractory to gamma-H2AX modification in yeast and mammals. *J. Cell Biol.* 178 209–218 10.1083/jcb.200612031 17635934 PMC2064441

[B16] Kitagawa R. Kastan M. B. (2005). The ATM-dependent DNA damage signaling pathway. *Cold Spring Harb. Symp. Quant. Biol.* 70 99–109 10.1101/sqb.2005.70.002 16869743

[B17] Kobayashi J. Tauchi H. Chen B. Burma S. Tashiro S. Matsuura S. (2009). Histone H2AX participates the DNA damage-induced ATM activation through interaction with NBS1. *Biochem. Biophys. Res. Commun.* 380 752–775 10.1016/j.bbrc.2009.01.109 19338747

[B18] Krawczyk P. M. Borovski T. Stap J. Cijsouw T. ten Cate R. Medema J. P. (2012). Chromatin mobility is increased at sites of DNA double-strand breaks. *J. Cell Sci.* 125 2127–2133 10.1242/jcs.089847 22328517

[B19] Kruhlak M. J. Celeste A. Dellaire G. Fernandez-Capetillo O. Muller W. G. McNally J. G. (2006). Changes in chromatin structure and mobility in living cells at sites of DNA double-strand breaks. *J. Cell Biol.* 172 823–834 10.1083/jcb.200510015 16520385 PMC2063727

[B20] Li J. Coic E. Lee K. Lee C. S. Kim J. A. Wu Q. (2012). Regulation of budding yeast mating-type switching donor preference by the FHA domain of Fkh1. *PLoS Genet.* 8:e1002630 10.1371/journal.pgen.1002630 PMC3320585 22496671

[B21] Lieberman-Aiden E. van Berkum N. L. Williams L. Imakaev M. Ragoczy T. Telling A. (2009). Comprehensive mapping of long-range interactions reveals folding principles of the human genome. *Science* 326 289–293 10.1126/science.1181369 19815776 PMC2858594

[B22] Lin C. Yang L. Tanasa B. Hutt K. Ju B. G. Ohgi K. (2009). Nuclear receptor-induced chromosomal proximity and DNA breaks underlie specific translocations in cancer. *Cell* 139 1069–1083 10.1016/j.cell.2009.11.030 19962179 PMC2812435

[B23] Lomvardas S. Barnea G. Pisapia D. J. Mendelsohn M. Kirkland J. Axel R. (2006). Interchromosomal interactions and olfactory receptor choice. *Cell* 126 403–413 10.1016/j.cell.2006.06.035 16873069

[B24] Matsuoka S. Ballif B. A. Smogorzewska A. McDonald E. R. III Hurov K. E. Luo J. (2007). ATM and ATR substrate analysis reveals extensive protein networks responsive to DNA damage. *Science* 316 1160–1166 10.1126/science.1140321 17525332

[B25] Meier A. Fiegler H. Munoz P. Ellis P. Rigler D. Langford C. (2007). Spreading of mammalian DNA-damage response factors studied by ChIP-chip at damaged telomeres. *EMBO J.* 26 2707–2718 10.1038/sj.emboj.7601719 17491589 PMC1888678

[B26] Panier S. Ichijima Y. Fradet-Turcotte A. Leung C. C. Kaustov L. Arrowsmith C. H. (2012). Tandem protein interaction modules organize the ubiquitin-dependent response to DNA double-strand breaks. *Mol. Cell* 47 383–395 10.1016/j.molcel.2012.05.045 22742833

[B27] Pankotai T. Bonhomme C. Chen D. Soutoglou E. (2012). DNAPKcs-dependent arrest of RNA polymerase II transcription in the presence of DNA breaks. *Nat. Struct. Mol. Biol.* 19 276–282 10.1038/nsmb.2224 22343725

[B28] Renkawitz J. Lademann C. A. Kalocsay M. Jentsch S. (2013). Monitoring homology search during DNA double-strand break repair in vivo. *Mol. Cell* 50 261–272 10.1016/j.molcel.2013.02.020 23523370

[B29] Rogakou E. P. Boon C. Redon C. Bonner W. M. (1999). Megabase chromatin domains involved in DNA double-strand breaks in vivo. *J Cell Biol.* 146 905–916 10.1083/jcb.146.5.905 10477747 PMC2169482

[B30] Rogakou E. P. Pilch D. R. Orr A. H. Ivanova V. S. Bonner W. M. (1998). DNA double-stranded breaks induce histone H2AX phosphorylation on serine 139. *J. Biol. Chem.* 273 5858–5868 10.1074/jbc.273.10.5858 9488723

[B31] Savic V. Yin B. Maas N. L. Bredemeyer A. L. Carpenter A. C. Helmink B. A. (2009). Formation of dynamic gamma-H2AX domains along broken DNA strands is distinctly regulated by ATM and MDC1 and dependent upon H2AX densities in chromatin. *Mol. Cell* 34 298–310 10.1016/j.molcel.2009.04.012 19450528 PMC2744111

[B32] Shanbhag N. M. Rafalska-Metcalf I. U. Balane-Bolivar C. Janicki S. M. Greenberg R. A. (2010). ATM-dependent chromatin changes silence transcription in cis to DNA double-strand breaks. *Cell* 141 970–981 10.1016/j.cell.2010.04.038 20550933 PMC2920610

[B33] So S. Davis A. J. Chen D. J. (2009). Autophosphorylation at serine 1981 stabilizes ATM at DNA damage sites. *J. Cell Biol.* 187 977–990 10.1083/jcb.200906064 20026654 PMC2806275

[B34] Soutoglou E. Misteli T. (2008). Activation of the cellular DNA damage response in the absence of DNA lesions. *Science* 320 1507–1510 10.1126/science.1159051 18483401 PMC2575099

[B35] Spilianakis C. G. Lalioti M. D. Town T. Lee G. R. Flavell R. A. (2005). Interchromosomal associations between alternatively expressed loci. *Nature* 435 637–645 10.1038/nature03574 15880101

[B36] Stewart G. S. Panier S. Townsend K. Al-Hakim A. K. Kolas N. K. Miller E. S. (2009). The RIDDLE syndrome protein mediates a ubiquitin-dependent signaling cascade at sites of DNA damage. *Cell* 136 420–434 10.1016/j.cell.2008.12.042 19203578

[B37] Stewart G. S. Wang B. Bignell C. R. Taylor A. M. Elledge S. J. (2003). MDC1 is a mediator of the mammalian DNA damage checkpoint. *Nature* 421 961–966 10.1038/nature01446 12607005

[B38] Stracker T. H. Petrini J. H. (2011). The MRE11 complex: starting from the ends. *Nat. Rev. Mol. Cell Biol.* 12 90–103 10.1038/nrm3047 21252998 PMC3905242

[B39] Stucki M. Jackson S. P. (2006). gammaH2AX and MDC1: anchoring the DNA-damage-response machinery to broken chromosomes. *DNA Repair (Amst.)* 5 534–543 10.1016/j.dnarep.2006.01.012 16531125

[B40] Tsukuda T. Fleming A. B. Nickoloff J. A. Osley M. A. (2005). Chromatin remodelling at a DNA double-strand break site in *Saccharomyces cerevisiae*. *Nature* 438 379–383 10.1038/nature04148 16292314 PMC1388271

[B41] You Z. Bailis J. M. Johnson S. A. Dilworth S. M. Hunter T. (2007). Rapid activation of ATM on DNA flanking double-strand breaks. *Nat. Cell Biol*. 9 1311–1318 10.1038/ncb1651 17952060

[B42] Zhang Y. McCord R. P. Ho Y. J. Lajoie B. R. Hildebrand D. G. Simon A. C. (2012). Spatial organization of the mouse genome and its role in recurrent chromosomal translocations. *Cell* 148 908–921 10.1016/j.cell.2012.02.002 22341456 PMC3320767

